# A contextual-based approach for sarcasm detection

**DOI:** 10.1038/s41598-024-65217-8

**Published:** 2024-07-04

**Authors:** Nivin A. Helal, Ahmed Hassan, Nagwa L. Badr, Yasmine M. Afify

**Affiliations:** Faculty of Computer and Information Sciences, Ain Shams University, Cairo, 11566 Egypt

**Keywords:** Computational science, Computer science, Software

## Abstract

Sarcasm is a perplexing form of human expression that presents distinct challenges in understanding. The problem of sarcasm detection has centered around analyzing individual utterances in isolation which may not provide a comprehensive understanding of the speaker’s sarcastic intent. Our work addresses this problem by exploring and understanding the specific contextual cues that contribute to sarcasm. In this paper, we propose an enhanced approach for sarcasm detection using contextual features. Our methodology involves employing pre-trained transformer models, RoBERTa and DistilBERT, and fine-tuning them on two datasets: the News Headlines and the Mustard datasets. Incorporating contextual information, the proposed approach yielded the best performance, achieving an impressive F1 score of 99% on News Headlines dataset and 90% on Mustard dataset. Moreover, we experimented summarizing the context into a concise short sentence. This enhancement reduced training time by 35.5% of the original time. We further validated the model trained on the News headlines dataset against the Reddit dataset, which resulted in 49% F1 score without context data. However, with the inclusion of context data, the F1 score surged to 75%. Proposed approach enhances the understanding of sarcasm in different contextual settings, enabling more accurate sentiment analysis and better decision-making in various applications.

## Introduction

Sarcasm, a form of communication characterized by irony and mockery, presents a unique challenge for natural language processing systems. Merriam Webster dictionary defines sarcasm as “the use of words that mean the opposite of what you really want to say especially in order to insult someone, to show irritation, or to be funny”^1^. Sarcasm has become a prevalent and versatile form of expression in various aspects of contemporary life. In casual conversations among friends or family, it often adds a layer of humor and depth. In social media, sarcasm stands out as one of the most common and effective figurative devices. The expanding landscape of markets, with many products and services receiving diverse public feedback, has given rise to the need for sarcasm detection and analysis. Additionally, sitcoms and TV shows exploring human experiences offer a rich canvas for sarcasm, offering valuable glimpses and insight into the thought processes and communication styles of specific groups or individuals. In the healthcare domain, sarcasm detection plays a role in uncovering mental health diagnoses, such as depression. Furthermore, understanding public sentiment regarding critical events within a political context adds another layer of significance to sarcasm analysis. Moreover, in the realm of online news, where information dissemination is rapid and diverse, sarcasm serves as a notable linguistic tool.

Sarcasm detection is pivotal in refining sentiment analysis applications, particularly when individuals express negative sentiments positively. The ability to identify sarcasm ensures a more accurate interpretation of the underlying sentiment conveyed in text. By distinguishing between the literal and intended meanings of statements, sarcasm detection models enhance the precision of sentiment analysis, allowing applications to discern subtle nuances that may be missed by traditional sentiment classifiers. This nuanced understanding is vital for capturing the true sentiment landscape, especially when individuals employ sarcasm as a means of expressing negativity or criticism in a more indirect and witty fashion.

Various strategies have been employed to address sarcasm in sentiment analysis^2–5^. Multimodal techniques, which consider diverse data modalities like text, image, and audio, have been created^6–8^. Successful alternatives involve modeling the speaker’s profile and taking into consideration their past expressions to enhance comprehension of conveyed meanings^9^. Furthermore, research has delved into approaches that construct the audience’s profile and assess its capacity to discern sarcasm^10,11^. These approaches utilize deep learning models, such as recurrent neural networks (RNNs), long short-term memory networks (LSTMs), and transformer-based models like bidirectional encoder representations from transformers (BERT). These models can capture complex semantic nuances in language, making them effective for sarcasm detection. Ensemble methods, which combine predictions from multiple models, have been explored for sarcasm detection, combining the strengths of different algorithms or features can improve overall performance and robustness. However, in sarcasm detection, understanding the temporal context of text has become essential. Models that incorporate information about the sequence of statements in a conversation have shown promising results. Recognizing that different domains exhibit distinct contextual patterns; some approaches focus on domain-specific context handling. This involves tailoring models to understand the unique linguistic nuances of specific contexts such as social media, news articles, or customer reviews.

Accurate detection of sarcasm is crucial for various applications. However, detecting sarcasm becomes even more intricate when considering the influence of contextual information, as it significantly affects the intended meaning of sarcastic statements. The contribution of the work is threefold: First, propose an approach that utilizes contextual data from news headlines and sitcoms to improve the accuracy of sarcasm detection. It aims to overcome the limitations of existing approaches by incorporating not only linguistic and syntactic features, but also rich contextual cues present in these domains. Second, minimize the training time by encapsulating the conversation context into a concise sentence focusing on the core meaning for efficient comprehension. Third, assess the effectiveness of the proposed approach through comprehensive experiments conducted on two popular datasets, and further validated on a third dataset.

This paper is organized into six sections. “Related work” section reviews the related work. “Proposed approach” section details the proposed approach. “Experimentation and evaluation” section presents the experimental and evaluation methods. “Discussion” section presents the discussion and comparison with existing research studies. Finally, “Conclusion and future work” section concludes with findings and future research directions.

## Related work

Sarcasm has been widely studied by researchers in linguistics, psychology, and natural language processing. In this section, we aim to curate and present a selection of the most impactful papers within this domain, organized into four subsections, each reflecting a different methodology.

### Machine and deep learning approaches for sarcasm detection

In this category, research primarily concentrates on discerning patterns from textual data without relying on transformer-based architectures or contextual cues.

In Ref.^9^, Nayak and Bolla evaluated different vectorization and machine learning models for detecting sarcastic headlines. They highlight the importance of detecting sarcasm in news articles, given the rise of false and manipulative news and the popularity of humorous articles. Through their experiments, the authors found that using Long Short-Term Memory (LSTM) yields better recall, accuracy, and F1 score for sarcasm detection in headlines.

In Ref.^12^, Barhoom, Abu-Nasser, and Abu-Naser tackled the challenge of detecting sarcasm in news headlines. They explored machine learning and deep learning approaches by evaluating 21 machine learning algorithms and one deep learning algorithm.

In Ref.^13^, Sharma et al. proposed a hybrid sentence embedding-based technique using an auto encoder to detect sarcasm in social media. The model combines various sentence embedding methods and considers text over images for multimedia content.

### Transformers in sarcasm detection

In this category, research primarily revolves around the application of transformer-based models, such as BERT, Distil-BERT, and RoBERTa, for sarcasm detection without relying on contextual cues.

In Ref.^14^, Savini et al. proposed strong baselines using BERT pre-trained language models. Additionally, they enhance their BERT models by fine-tuning them on related intermediate tasks such as sentiment classification and emotion detection, using the correlation between sarcasm and implied negative sentiment and emotions. Experimental results on three distinct datasets demonstrate that their BERT-based models outperform previous approaches in sarcasm detection.

In Ref.^15^ Jayaraman et al. introduced a supervised learning approach for sarcasm detection using the “New Headlines” dataset. They conducted experiments with six supervised learning models: Naïve Bayes-support vector machine, logistic regression, bidirectional gated recurrent units, Bidirectional encoders representation from Transformers (BERT), Distil-BERT, and RoBERTa. Their findings suggested that RoBERTa outperformed the other models in terms of accuracy, indicating its effectiveness in sarcasm detection.

In Ref.^16^, Abaskohi et al. presented their methodology and results for sarcasm detection in the SemEval-2022 shared task 6. They tested different models and utilized data augmentation techniques.

In Ref.^17^, Sharma et al. introduced a novel hybrid ensemble model for sarcasm detection, specifically designed for social media datasets. They conducted experiments on three diverse datasets: “News Headlines”, the “Self-Annotated Reddit Corpus” (SARC), and the Twitter app. The proposed approach by Ref.^17^ involved utilizing fuzzy evolutionary logic and ensemble embedding from multiple models, namely Word2Vec, Glove, and BERT. By combining these techniques, the authors aimed to enhance the accuracy of sarcasm detection across social media platforms.

### Contextual approaches (non-transformer) in sarcasm detection

In this category, research studies utilizing contextual information, without relying on transformer architectures are presented.

In Ref.^18^, Zhang et al. introduced CFN, a Complex-Valued Fuzzy network for sarcasm detection in conversations. CFN applies quantum theory and fuzzy logic to address language vagueness and uncertainty. The model treats the target utterance as a quantum superposition of words and captures contextual interactions. A density matrix represents vagueness and uncertainty, and a quantum fuzzy measurement yields probabilistic outcomes for sarcasm recognition. CFN outperformed strong baselines on the Mustard and Reddit datasets, demonstrating its effectiveness in sarcasm detection.

In Ref.^19^, Băroiu and Trăușan-Matu conducted a comparison of deep learning models for automatic detection of sarcasm context. Their aim was to identify models that can accurately detect the contexts in which sarcasm occurs or is appropriate. Among the models evaluated, an attention-based long short-term memory (LSTM) architecture emerged as the top performer.

### Transformers using context in sarcasm detection

This category focuses specifically on approaches that combine transformer architectures with contextual information for sarcasm detection. By incorporating both transformer-based language understanding and contextual cues from the surrounding text, these methods achieve high accuracy in identifying sarcastic expressions.

In Ref.^20^, Jaiswal presented a deep neural architecture for sarcasm detection in social media platforms like Reddit and Twitter. They investigated various pre-trained language representation models (PLRMs) such as BERT and RoBERTa, and fine-tuned them on the Twitter dataset. By considering the contextual information from the previous three most recent utterances.

In Ref.^21^, Baruah et al. focused on sarcasm detection in the context of conversational data. The authors utilized BERT, BiLSTM, and SVM classifiers for the task. They experimented with different amounts of contextual information, ranging from no context to including the last one, two, or three utterances, as well as all utterances. The results showed that incorporating the last utterance along with the response improved the classifier’s performance for the Twitter dataset. For the Reddit dataset, using only the response without contextual information yielded the best performance.

In addressing a notable gap in previous research efforts, an examination of existing sarcasm detection literature revealed a critical shortage in the focus on context data. Notably, a considerable number of studies did not adequately account for contextual information. While some papers did incorporate context, a significant portion did not thoroughly analyze its impact on sarcasm detection, leading to limitations in sarcasm detection accuracy. This prompted innovating an enhanced approach that prioritizes context. In the News Headlines dataset, the context was absent, thus collecting and integrating contextual data was crucial to addressing the gap in the previous research efforts.

## Proposed approach

**Figure 1:**
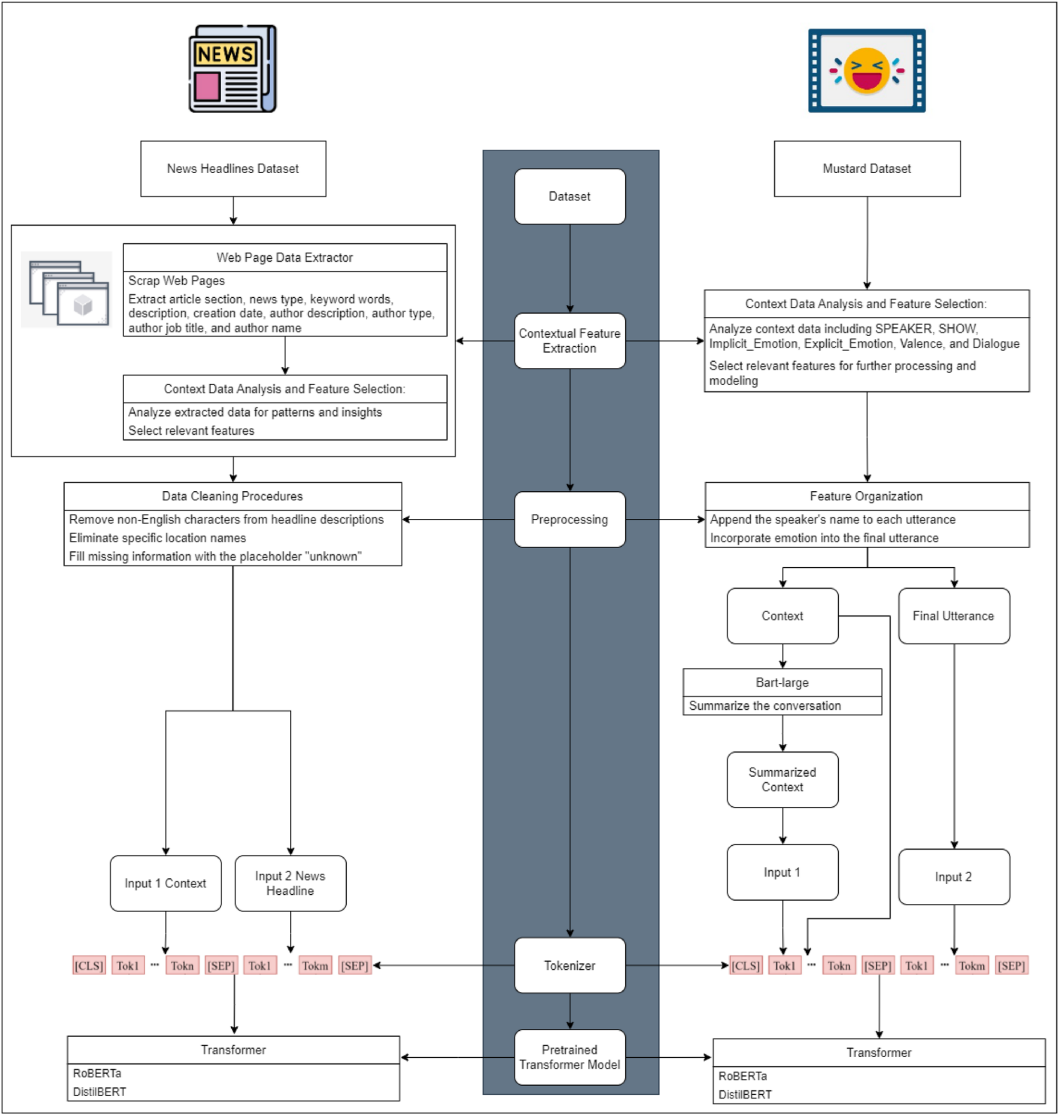
Proposed sarcasm detection approach workflow.

In the proposed sarcasm detection approach, we prioritize the pivotal role of contextual information, recognizing its universal significance across diverse datasets. The proposed approach adopts a dual focus: firstly, a meticulous analysis of the impact of contextual information on sarcasm, acknowledging its pivotal role in refining sarcasm detection accuracy. Secondly, we collect context data, ensuring adaptability where such information is not available to enrich the understanding of sarcasm, subsequently, its detection. To implement this approach, we employ the Robustly Optimized BERT Approach (RoBERTa) architecture, fine-tuned with sarcasm detection task. The workflow for the proposed sarcasm detection approach is presented in Fig. 1 and is thoroughly described in the following subsections.

### Datasets

Two popular datasets, namely the Mustard dataset^22^ and the News Headlines dataset^23^ were employed. These two datasets differ significantly in their nature and size, with the Mustard dataset highlighting scenes from comedy TV shows, while the News Headlines dataset revolves around online news headlines, encompassing both satirical and non-sarcastic content. Further details about the datasets are provided in Table 1.

1. Mustard Dataset is a collection of comedy TV show scenes, featuring popular sitcoms like Friends and The Big Bang Theory. It encompasses scenes, each of which contains multiple lines of dialogue.
2. News Headlines Dataset is primarily centered around online news headlines. It is sourced from two distinct news websites. The first website, “The Onion”, specializes in creating satirical versions of current events, providing a platform for sarcastic news headlines. On the other hand, the dataset also includes real news headlines obtained from the “HuffPost” news website, which focuses on delivering non-sarcastic news content.

**Table 1:** Mustard and News Headlines datasets details.

Dataset	Content	Columns	Number of records	Contextual information
Mustard dataset	Comedy TV Shows	Sentence, dialogue, and implicit emotion	1202	Scene dialogue, speaker, and implicit emotion
News Headlines dataset	Online News	Headline and article link	26,709	None

### Contextual feature extraction module

Contextual feature extraction is crucial in the sarcasm field as it captures the surrounding dialogue, enabling a deeper understanding of linguistic nuances and enhancing the ability to identify and interpret sarcastic expressions within diverse datasets.

In the News Headlines dataset, we took measures to enhance the contextual information. This involved gathering additional data by scraping through the article links, which enabled us to obtain valuable details such as the author’s name, the article section (e.g., politics, business), and a description of the headline. For the news headlines dataset, we further investigated the inclusion of the article section as part of the input. Table 2 demonstrates the distribution of sarcastic labels across different article sections in the dataset. We observed a balanced distribution for approximately one-third of the dataset, where the number of sarcastic instances was comparable to the number of non-sarcastic instances. However, it is important to note that the remaining two-thirds of the dataset exhibited an unbalanced distribution, with varying proportions of sarcastic and non-sarcastic data.

**Table 2:** Ratio of sarcastic and non-sarcastic sentences in sample of article sections with highest count of records in News Headlines dataset.

Article section	Number of sarcastic records	Ratio (%)	Number of not sarcastic records	Ratio
Business	389	47	445	53%
Entertainment	1111	43	1458	57%
Politics	1583	32	3388	68%
news	1520	100	0	None
Local	2715	100	0	None

In the Mustard dataset, it had already been equipped with pertinent features, including scene details, dialogue, and implicit emotions. Exploring the impact of including speaker names on the sarcasm detection task, we observed a balanced distribution of sarcastic and non-sarcastic sentences across different speakers. This balance is evident in Table 3, where each person exhibits a similar number of sarcastic and non-sarcastic sentences. To incorporate contextual information in the Mustard dataset, we merged the entire scene dialogue with the potentially sarcastic sentence and included implicit emotions in the input data. This step aimed to provide a comprehensive context for the dataset.

**Table 3:** Ratio of sarcastic and non-sarcastic sentences for sample actor with highest count of records in Mustard dataset.

Actor	Number of sarcastic scenes	Ratio (%)	Number of not sarcastic scenes	Ratio (%)
AMY	26	48	28	52
BERNADETTE	26	49	27	51
CHANDLER	119	76	38	24
DOROTHY	38	97	1	3
GILFOYLE	23	50	23	50
HOWARD	68	50	69	50
JOEY	3	8	33	92
LEONARD	59	52	55	48
MONICA	6	21	23	79
OTHER	18	50	18	50
PENNY	72	57	55	43
PERSON	12	27	33	73
PHOEBE	8	23	27	77
RACHEL	7	23	24	77
RAJ	22	40	33	60
ROSS	5	13	33	87
SHELDON	61	48	65	52

### Preprocessing module

In the context of sarcasm detection, incorporating additional contextual information into the input is crucial to enhance the model’s understanding and improve its performance. However, the process of adding context data requires careful consideration, as the goal is to enable the model to accurately identify sarcastic sentences.

In the News Headlines dataset, to ensure data cleanliness, we performed cleaning procedures on the headline description. This included removing non-English characters and specific location names associated with the news event, such as “LITTLE ROCK” and “WASHINGTON”. By eliminating these elements, we aimed to maintain consistency and improve the quality of the dataset. In instances where information was missing, we addressed the gaps by filling them with the placeholder “unknown”. This approach allowed us to maintain data integrity and handle any missing values appropriately.

As part of our approach, we enhanced the input for the News Headlines dataset by adding the description with a context separator. By cleaning the description from any potentially misleading elements, we aimed to ensure that the model’s sarcasm detection capabilities were solely dependent on the context at hand. This way, the model can better understand the context and accurately identify instances of sarcasm based on the meaning of the context.

**Figure 2:**
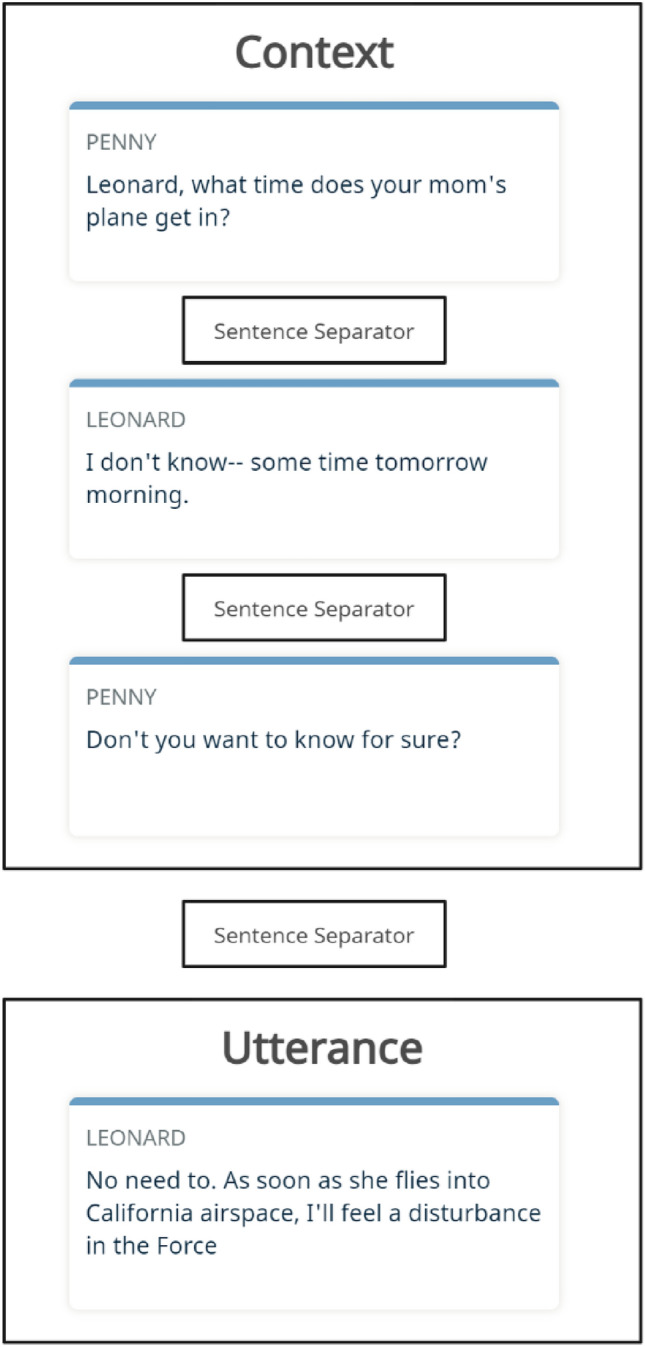
Sample utterance with its context in Mustard dataset.

In the Mustard dataset, we addressed this challenge by including the speaker’s name for each sentence. This approach was adopted to facilitate the model’s comprehension of the dialogue, as speakers often have multiple sentences. Additionally, we used a separator token between sentences to aid RoBERTa model in processing the input effectively (as shown in Fig. 2).

**Figure 3:**
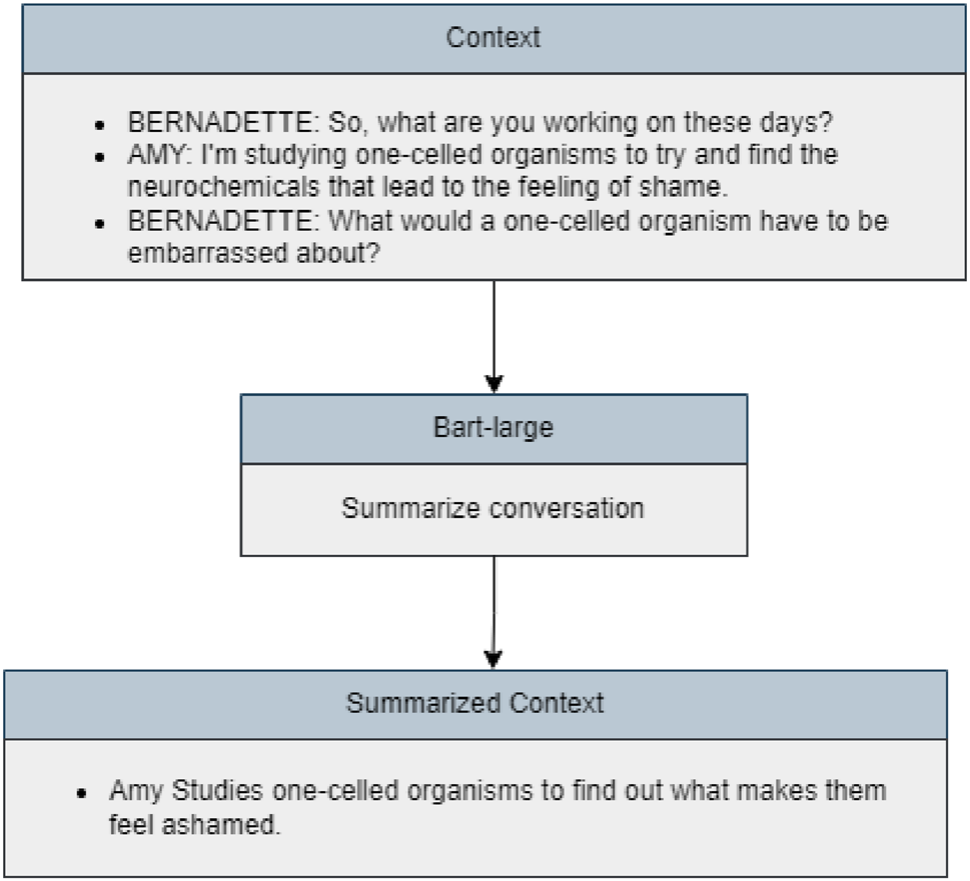
Conversation summarization with Bart-large.

In our investigation, we further explored the conversion of contextual conversations into summarized forms to generalize the input shape. Text summarization aids researchers by distilling essential semantic cues from voluminous textual data, facilitating the development of robust classification models, aiding in the identification of key sarcastic cues facilitating efficient data processing, enhancing model interpretation, promoting generalization and ensuring adaptability. Employing a model known as MEETING_SUMMARY^24^ constructed with Bart-large, developed by Karthick Kaliannan Neelamohan, we summarized conversations into concise, meaningful sentences containing the main points (as shown in Fig. 3).

### Tokenizer module

The input data is tokenized and encoded using the RoBERTa Tokenizer from the Hugging Face library^25^. This process involves applying truncation and padding to ensure the inputs have consistent lengths.

### Pretrained model module

The RoBERTa model architecture comprises 12 transformer encoder layers. Each encoder layer employs self-attention mechanisms and feed-forward neural networks to process the input sequences and capture contextual information. On top of the RoBERTa encoder layers, we add a classification head for sarcasm detection. To compute the loss during training, we utilize the “CrossEntropyLoss” function.

## Experimentation and evaluation

### Experimental setup

Experiments were conducted on Google Colab’s T4 GPU. The T4 GPU offers high-performance computing capabilities, enabling faster model training and evaluation compared to traditional CPU setups.

The input parameters used in the training and evaluation processes are listed in Table 4. To assess the performance of the proposed approach, we divided the data into training and testing sets, allocating 80% for training and 20% for testing.

**Table 4:** Model input parameters.

Parameter	Train batch size	Learning rate	Epochs	Evaluation batch size	Weight decay
Values	32	5e−5	5	64	0.01

### Evaluation metrics

The most popular metric for calculating a model’s performance is accuracy, which gives a good idea about performance in the case of balanced classes^15^ and F1 score as it uses a harmonic medium to make end values even more extreme^15^.

1 $$\text{Accuracy}=\frac{TruePositive+TrueNegative}{TruePositive+FalsePositive+TrueNegative+FalseNegative},$$

2 $$\text{Precision}=\frac{TruePositive}{TruePositive+FalsePositive},$$

3 $$\text{Recall}=\frac{TruePositive}{TruePositive+FalseNegative},$$

4 $$\text{F}1\text{ Score}=2\cdot \frac{\mathit{Pr}ecision\times Recall}{\mathit{Pr}ecision+Recall}.$$

### Experimental results

The main objective of experiments is to analyze the impact of incorporating contextual data on enhancing sarcasm detection accuracy. To achieve this goal, two experiments are conducted using RoBERTa. Experiment 1 is performed on the Mustard dataset, while experiment 2 is performed on the News Headlines dataset. On another note, we examine the performance of DistilBERT in comparison to RoBERTa in experiment 3. Additionally, to further assess the proposed approach, validation is conducted on the Reddit dataset^26^ in experiment 4.

#### Experiment 1

We evaluate the effectiveness of using only the sentence compared to including additional information like implicit emotion and scene dialogue sentences, using Mustard dataset. The results are presented in Table 5. In the first experiment, we solely rely on the sentence, which achieves low accuracy. This indicates that sarcasm cannot be accurately determined based solely on the sentence unless it is explicitly and unequivocally sarcastic. However, when we introduce dialogue and emotion into the input, the accuracy improves. Nevertheless, we encounter a problem when names are not used in the dataset, which can be caused by three main reasons:

1. The proposed approach can comprehend conversations, but there might be multiple sequential sentences attributed to the same person. This ambiguity can pose challenges in accurately identifying sarcasm.
2. There might be more than two speakers, the model should identify them.
3. The proposed approach can acquire knowledge from the speakers, understanding how they tell jokes. This aspect proves beneficial for predicting sarcasm in comedy shows. It is important to note that the dataset is balanced, meaning that each speaker has a similar number of sarcastic and non-sarcastic records.

**Table 5:** Proposed approach accuracy on Mustard dataset.

Attempt	Features used	Accuracy	F1 score
1	Sentence only	0.56	0.46
2	Dialogue without speakers’ names—emotion	0.64	0.50
3	Dialogue with speakers’ names	0.74	0.69
4	Dialogue with speakers’ names—emotions	0.89	0.90
5	Summarized conversation—emotions	0.868	0.877

Building upon the previous findings, we further experiment by incorporating the speaker information into the dialogue. This addition resulted in higher accuracy compared to the previous attempts. By including the speakers, we were able to better understand the context and improve the model’s ability to predict sarcasm. In our fourth attempt, we combined both emotions and dialogues in the input data. This combination led to a substantial increase in F1 score by 44% compared to using the final utterance only, by 40% compared to incorporating dialogue without speaker names and by 21% compared to attempt without using emotions.

Finally, exploring the impact of summarization compared to inserting the entire conversation in the fifth attempt. Experimental results show that the training time is reduced by 35.5%, indicating the superiority of this approach in terms of time efficiency. Notably, comparable accuracy and F1 score are achieved.

Overall, these experiments demonstrate the importance of contextual data, including implicit emotions, scene dialogues, and speaker information, in accurately predicting sarcasm. The combination of these elements resulted in improved accuracy, highlighting the significance of considering a broader context when analyzing sarcasm in the Mustard dataset.

#### Experiment 2

We seek to determine the most effective combination of contextual information, incorporating the News Headlines dataset in order to achieve high accuracy in sarcasm detection.

During our experimentation, it was found that the model achieves the highest accuracy when both the description and article section were utilized. However, it is worth noting that the description’s inclusion resulted in longer processing times compared to using only the article section. Furthermore, it was observed that incorporating the author’s name also leads to a noticeable increment in accuracy. By including this additional contextual detail, the model gains further insights that contribute to its ability to accurately identify sarcasm in the headlines (as shown in Table 6). Adding the description contextual information improves F1 score by 9% compared to not adding any context at all. However, compared to including only the author’s name, the F1 score has increased by 6%.

**Table 6:** Proposed approach accuracy on News Headlines dataset.

Attempt	Meta data	Accuracy	F1 score
1	Without meta data	0.91	0.90
2	Author name	0.94	0.93
3	Article section	0.99	0.99
4	Description	0.99	0.99

To ensure that the proposed approach is not overfitting the data, we compare the training loss and validation loss for each epoch. Table 7 illustrates that the validation loss closely tracks the training loss, indicating that the proposed approach generalizes well and is not overfitting to the training data. Additionally, the evaluation results (shown in Table 8) reinforce this conclusion, further validating the approach’s ability to accurately detect sarcasm without suffering from overfitting issues.

**Table 7:** Proposed approach training results on News Headlines dataset.

Training loss	Validation loss	Accuracy	F1 score
0.154	0.0268	0.9956	0.995
0.033	0.0145	0.9970	0.996
0.013	0.0152	0.9977	0.997
0.008	0.0122	0.9977	0.997
0.002	0.0239	0.9964	0.995
0.001	0.0150	0.9981	0.997

**Table 8:** Proposed approach evaluation results on News Headlines dataset.

Evaluation loss	Evaluation accuracy	Evaluation F1 score	Evaluation runtime	Epochs
0.0122	0.997	0.997	60.3882	5

Overall, the experiment demonstrated that incorporating contextual data, specifically the description, article section, and author name, significantly improved the accuracy of sarcasm detection in the News Headlines dataset.

#### Experiment 3

Experiments 1 and 2 comprised evaluating RoBERTa across different scenarios. In contrast, in this experiment, we investigate DistilBERT, recognized as a lighter and faster variant of the BERT model. We aim to gauge its performance in terms of time efficiency and compare it with RoBERTa. In Tables 9 and 10, we conducted a comparison between DistilBERT and RoBERTa using the News Headlines dataset and the Mustard datasets respectively. A significant discrepancy in training time is observed for DistilBERT, despite its accuracy and F1 score being nearly identical to those of RoBERTa. In the News Headlines dataset, DistilBERT outperforms RoBERTa with a 52% reduction in training time of the RoBERTa duration. Similarly, in the Mustard dataset, DistilBERT surpasses RoBERTa, achieving a 56.1% reduction in training time relative to the RoBERTa duration when using dialogue with speakers’ names and emotions. Additionally, when using dialogue with speakers’ names and emotions, while omitting summarization, DistilBERT achieves a 51.8% reduction in processing time compared to the RoBERTa duration.

**Table 9:** Results of the proposed approach on the News Headlines dataset comparing RoBERTa with DistilBERT.

Model	Context data	Validation loss	Accuracy	F1 score	Training time
RoBERTa	Headline description	0.0122	0.997	0.997	1 h and 29 min
DistilBERT	Headline description	0.0173	0.996	0.995	42 min and 15 s

**Table 10:** Results of the proposed approach on the Mustard dataset comparing RoBERTa with DistilBERT.

Model	Context data	Validation loss	Accuracy	F1 score	Training time
RoBERTa	Dialogue with speakers’ names—emotions	0.237	0.89	0.90	3 min and 48 s
DistilBERT		0.24	0.896	0.90	1 min and 40 s
RoBERTa	Summarized conversation—emotions	0.298	0.868	0.877	2 min and 27 s
DistilBERT		0.296	0.87	0.877	1 min and 11 s

#### Experiment 4

To further assess the performance of the proposed model, we conducted a validation experiment using the Reddit (SARC) dataset, which originally comprises approximately 1.3 million comments, including parent comments and associated subreddits, all labeled for sarcasm. For our study, we utilized a preprocessed version of the Reddit dataset, which underwent cleaning and standardization procedures by Frankle Muchahary’s project^24^. This preprocessing involved removing NA values, converting text to lowercase, eliminating special characters and numbers, removing stop words, and lemmatization. For the validation process, we curated a subset of 7370 records from the preprocessed Reddit dataset. Contextual information, including parent comments, and subreddits, was employed during the validation process. The proposed model was trained on news headline dataset twice: once without incorporating metadata and another time with enhanced metadata. Upon training completion, the model’s performance was evaluated on the Reddit validation dataset using both variations. Without the additional metadata, the model exhibited an accuracy of 0.495 and an F1 score of 0.55. However, with the inclusion of metadata during training, substantial performance improvements were observed. The accuracy rose to 0.608, and the F1 score increased significantly to 0.75. These validation results underscore the crucial role of integrating contextual data in sarcasm detection models. Moreover, the successful validation of the proposed model on the Reddit dataset validates its ability to generalize across diverse datasets, affirming its robustness and reliability in real-world applications.

## Discussion

It is notable that models tailored to specific contexts often outperform conventional methods. This approach enables models to grasp the entirety of meaning rather than relying solely on individual words or characters. For instance, to the best of the authors knowledge, we are the sole collectors of contextual or metadata for the News Headlines dataset. The proposed model trained on this unique data achieved unparalleled accuracy surpassing prior benchmarks, as demonstrated in Table 11. Moreover, in both News Headlines and Mustard datasets, we meticulously analyzed how the context affects model performance and accuracy. Furthermore, in Mustard dataset, we adopted a strategy of converting inputs into succinct contextual sentences, enhancing the model’s ability to generalize and focus solely on pertinent details. Comparison with other detection methods is shown in Table 12.

**Table 11:** Comparison of the proposed approach with existing methods on News Headlines dataset.

Research work	Word embedding	Model	Accuracy	F1 score
^9^	BERT	LSTM	89.5	89.7
^12^	GloVe	LSTM	95.27	95.37
^13^	LSTM, BERT and USE	SoftMax	90.8	NA
^15^	Word2Vec, GloVe, and fastText	RoBERTa-Base	NA	93.11
Proposed approach	RoBERTa Tokenizer	RoBERTa-Base	99.7	99.7

**Table 12:** Comparison of the proposed approach with existing methods on Mustard dataset.

Research work	Word embedding	Model	Accuracy	F1 score
^19^	GloVe	Attn–LSTM	NA	60.1
^18^		CFN	75.4	75.4
Proposed approach	RoBERTa Tokenizer	RoBERTa-Base	89	90

Using the News Headlines dataset, the proposed approach outperformed work in Ref.^9^ by 10%, Ref.^12^ by 4.33% and Ref.^15^ by 6.59 in F1 score and Ref.^13^ by 8.9% in accuracy. However, using the Mustard dataset, the proposed approach surpassed work in Ref.^19^ by 29.9%, and Ref.^18^ by 14.6% in F1 score.

## Conclusion and future work

Through extensive evaluation and analysis, it is evident that there is a clear correlation between the inclusion of context data and improved sarcasm detection accuracy. In Mustard dataset, augmenting the model input with implicit emotion and full dialogue resulted in a noticeable progression in accuracy levels. This substantiates the premise that context plays a pivotal role in sarcasm detection, as evidenced by the improved performance on a dataset rich in contextual cues. Additionally, it is worth noting that we condensed the contextual conversation, resulting in a substantial reduction in the processing time. Extending the investigation to another domain, experiments on the News Headlines dataset further validated the efficacy of the proposed approach. Integrating metadata, article sections, descriptions, and author names into the model input consistently led to higher accuracy, emphasizing the efficiency and applicability of the proposed approach across diverse datasets. The efficacy of the proposed approach was further assessed by validation using the Reddit (SARC) dataset.

The adoption of the RoBERTa and DistilBERT models, chosen for their proficiency in understanding context, proved crucial in achieving the observed improvements in accuracy. The models ability to capture nuanced contextual information aligned seamlessly with the objectives of this study. We demonstrate the superiority of our proposed approach in terms of accuracy and F1 score. By incorporating context data from news headlines and sitcoms, our research contributes to the advancement of sentiment analysis and opinion mining in diverse domains.

In the future work, we will explore the synergy among textual, visual, and auditory cues to formulate more holistic models. By encompassing a spectrum of communicative modalities, these models aim to effectively capture the intricacies of sarcasm across various contexts, thereby contributing to a more comprehensive understanding of nuanced expressions.

## Data Availability

The News Headlines dataset analysed during the current study is available in the News Headlines Dataset For Sarcasm Detection repository, https://www.kaggle.com/datasets/rmisra/news-headlines-dataset-for-sarcasm-detection. The Mustard dataset analysed during the current study is available in the MUStARD_Plus_Plus repository, https://github.com/cfiltnlp/MUStARD_Plus_Plus. The Reddit (SARC) dataset analysed during the current study is available in the Sarcasm on Reddit repository, https://www.kaggle.com/datasets/danofer/sarcasm.
